# ZnO Nanomaterials: Current Advancements in Antibacterial Mechanisms and Applications

**DOI:** 10.3389/fchem.2020.00580

**Published:** 2020-07-21

**Authors:** Shengjie Jiang, Kaili Lin, Ming Cai

**Affiliations:** ^1^Department of Oral and Cranio-Maxillofacial Surgery, Shanghai Ninth People's Hospital, College of Stomatology, Shanghai Jiao Tong University School of Medicine, Shanghai, China; ^2^National Clinical Research Center for Oral Diseases, Shanghai, China; ^3^Shanghai Key Laboratory of Stomatology and Shanghai Research Institute of Stomatology, Shanghai, China

**Keywords:** ZnO nanomaterials, antibacterial activity, mechanism, applications, review

## Abstract

The prevalence of various diseases caused by bacteria has been increasing, and some traditional antibiotics have been reported to have varying degrees of resistance. ZnO nanomaterials (ZnO-NMs), due to their excellent broad-spectrum antibacterial properties, lasting antibacterial effects, and excellent biocompatibility, have quickly become the research focus of new antibacterial agents. While the narrow light response range of ZnO-NMs has limited the antibacterial performance to some extent and modifying it by various means to improve its response under visible light, such as doping metal/non-metal atoms, depositing noble metals and coupling carbon materials, which is a new research hotspot. Herein, the current mainstream claims about the antibacterial mechanisms and applications of ZnO-NMs are reviewed.

## Introduction

Till now, the bacterial infection is still an urgent problem to be solved. The emergence and development of antibiotics have provided a simple and effective treatment for complex and severe diseases. However, the widespread production and abuse of antibiotics in recent decades have led to some “super bacteria” that have apparent resistance to antibiotics. Conventional antibiotic treatment has little effect on the increase of such bacteria.

Recently, nanotechnology, especially some nanomaterials with antibacterial activity such as metal nanoparticles, metal oxide nanoparticles, and carbon nanotubes, are considered as a new defense method against bacterial infections. The nanoscale surface effect and small size effect give these materials a unique antibacterial mechanism, mainly including three viewpoints: generate reactive oxygen species (ROS) or release metal ions to destroy bacterial DNA and protein; nanoparticles gather on the surface of the bacterial cell membrane and destroy the cell membrane (Qi et al., 2020) and interrupt transmembrane electron transfer (Li et al., 2008).

Compared with other antibacterial materials, as a traditional wide bandgap semiconductor, ZnO possesses excellent biocompatibility, safety, and long-term effectiveness, making it suitable for various biomedical applications (Liu et al., 2019). Moreover, the nanoscale gives it a unique antibacterial mechanism and significant antibacterial potential. ZnO nanoparticles (NPs) show significant bactericidal potential against various Gram-positive bacteria and Gram-negative bacteria such as Escherichia coli, Staphylococcus aureus, Pseudomonas aeruginosa, and Klebsiella pneumoniae (Luo et al., 2013). However, knowledge of the exact antibacterial mechanism of ZnO-NPs is still limited, and a correct understanding of its antibacterial mechanism is a prerequisite for effectively exerting its antibacterial potential. First, we review the latest research progress of the antibacterial mechanism of ZnO-NPs and the factors affecting its antibacterial activity. Then the applications of ZnO-NPs in antibacterial and antifungal are summarized.

## Antibacterial Mechanism of ZnO-NPs

### Generation of Reactive Oxygen Species (ROS)

**Figure 1:**
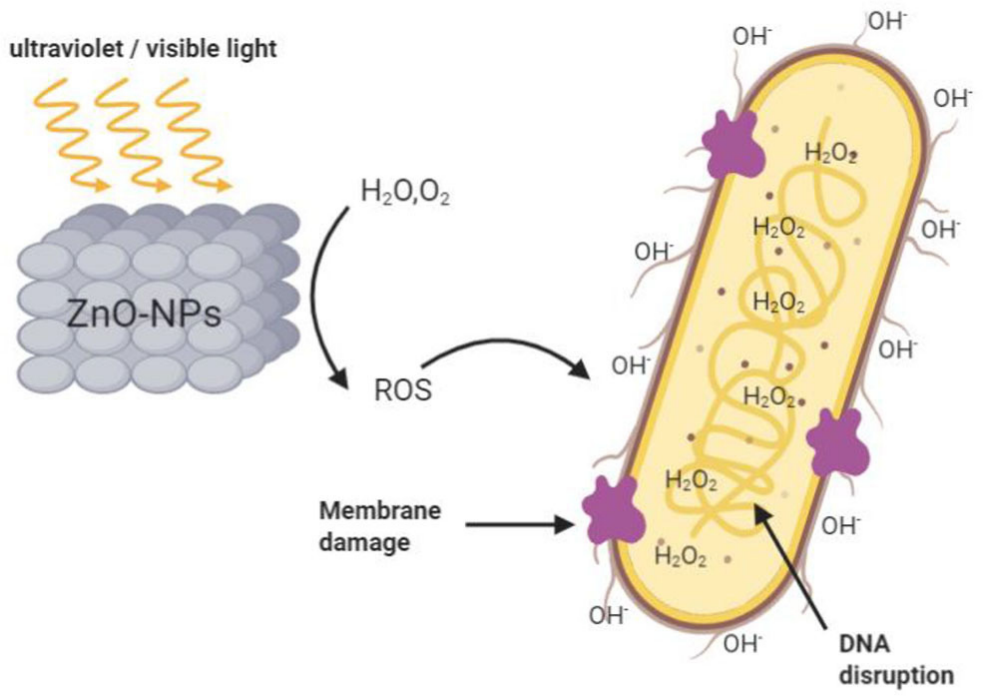
Mechanism of ROS production from ZnO-NPs and their bactericidal effect.

Reactive oxygen species (ROS) is the most common and widely accepted mechanism for the antibacterial activity of ZnO-NPs (Kumar et al., 2017). Active oxygen is a type of single-electron reduction product of oxygen, such as superoxide anion O^2−^, hydroxyl radical OH^−^ and hydrogen peroxide H_2_O_2_. ZnO is a wide bandgap semiconductor material. The electrons (e^−^) in its valence band transition under ultraviolet/visible light, leaving positively charged holes (H^+^). e^−^ and H^+^ undergo a series of redox reactions with oxygen and water on the surface of ZnO particles to generate ROS with extreme chemical activity (Miao et al., 2017; Figure 1).

**Figure 2:**
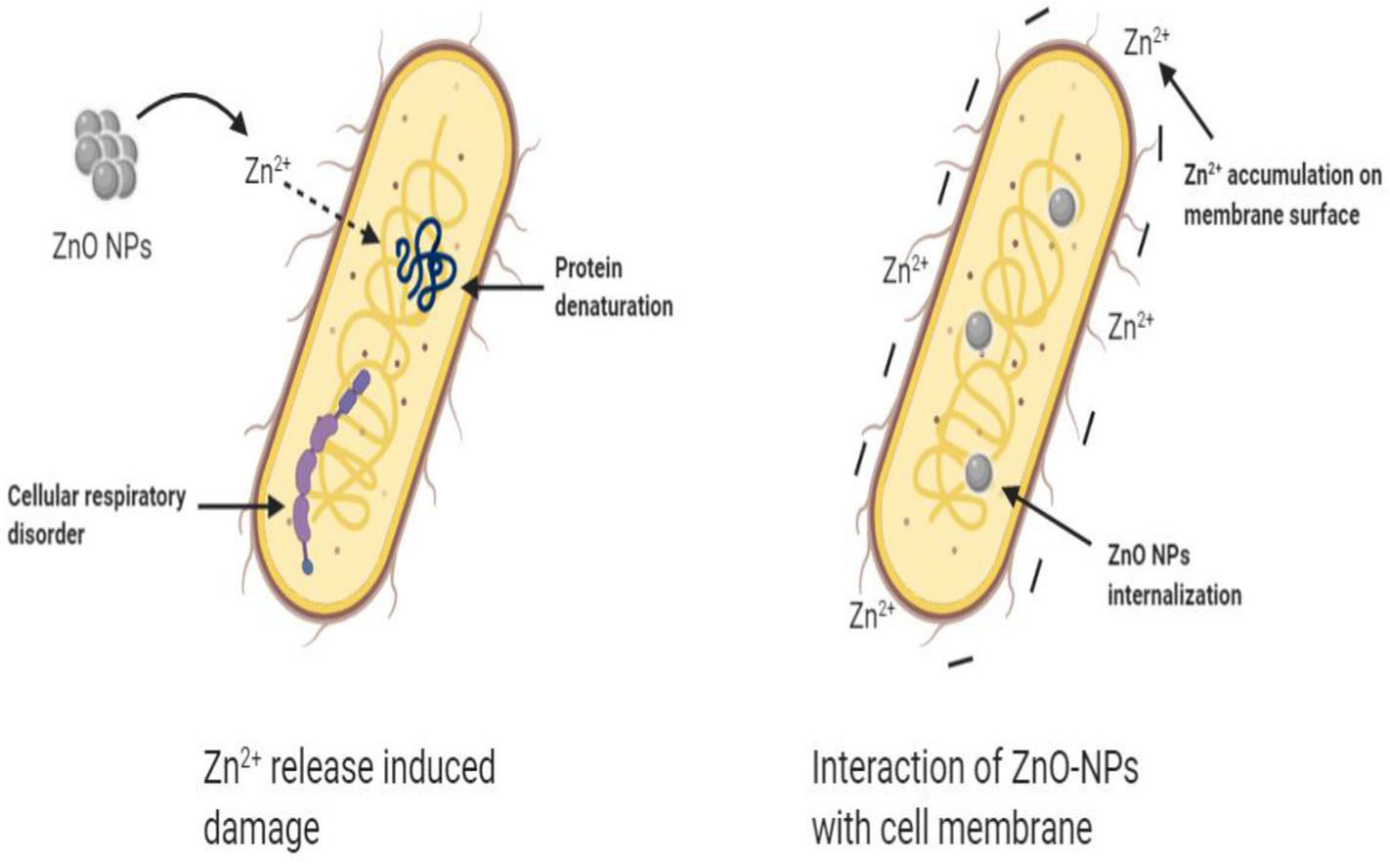
Two other antibacterial mechanisms of ZnO NPs.

ROS can cut off the chemical bonds of bacteria's organic matter, to achieve the bactericidal effect. Among them, the negatively charged peroxide cannot pass through the cell membrane, OH^−^ aggregates on the surface of bacterial cell membranes and causes cell membrane destruction, and H_2_O_2_ can penetrate the cell membrane, causing damage to the cell membrane and the destruction of DNA and protein in the membrane, which plays a bactericidal role (Kumar et al., 2017). However, it is worth noting that some studies have also found that ZnO also shows prominent antibacterial ability in the dark, and it is more significant when the bacterial concentration is low (Leung et al., 2016). In the study of Jeong et al. (2020) an array of ZnO nanorods was prepared by hydrothermal method and wrapped with an atomic layer of aluminum oxide to reduce the generation of active oxygen and the release of Zn^2+^. The results showed that in the dark, the mechanical damage to the cell membrane of *E. coli* accounted for 56.4% of the sterilization efficiency, while the chemical damage caused by the generation of reactive oxygen species and the release of Zn^2+^ accounted for only 37.8%. Hirota et al. (2010) also demonstrated that ZnO NPs have sustainable antibacterial activity against *E. coli* in the absence of light. These results indicate that in the absence of light, there may be additional mechanisms for generating active oxygen or other antibacterial mechanisms not related to light, as shown in Figure 2.

### Zn^2+^ Release Induced Damage

This theory holds that ZnO can slowly release Zn^2+^ in aqueous solution. Zn^2+^ can penetrate through the cell membrane and result in protein denaturation and loss of cell proliferation. Besides, Zn^2+^ can also destroy the electron transport system, leading to a cellular respiratory disorder. Joe et al. (2017) studied the antibacterial effects of various ZnO-NPs with different particle sizes and the number of oxygen vacancies in the dark state, and found that ZnO and Zn^2+^ adsorbed on the cell surface were the primary mode of action of antibacterial.

Although some studies attribute the antibacterial potential of ZnO to the release of Zn^2+^, no significant improvement in antibacterial effect was obtained with the increasing of Zn^2+^ concentration (Sawai, 2003). In the antibacterial experiment of Elena et al. (2016) on ZnO nanorod-modified graphene nanosheets (ZNGs) against Streptococcus mutans, the negligible dissolution of Zn^2+^ was observed, indicating high cell mortality in suspension is not related to Zn2 + release. It indicates that the release of Zn^2+^ should not be the primary mechanism of ZnO antibacterial.

### Interaction of ZnO-NPs With the Cell Membrane

The Interaction of ZnO-NPs with bacteria and subsequent destruction of the bacterial surface has been proposed to explain the antibacterial activity of ZnO-NPs. This mainly includes membrane dysfunction caused by the accumulation of positively charged Zn^2+^ on the surface of the cell membrane and the disorder of energy metabolism of bacterial substances caused by the internalization of ZnO-NPs.

#### Membrane Dysfunction

As shown in Figure 2, some studies suggest that Zn^2+^ will be electrostatically attracted to the negatively charged bacterial cell membrane surface, thereby interfering with the charge balance on the cell membrane surface, resulting in severe cell deformation, and finally leading to bacterial lysis (Wang et al., 2014). Zhang et al. (2007) showed that ZnO-NPs caused damage to the cell membrane of *E. coli*, and further research found that this damage may be caused by the direct interaction between ZnO-NPs and the cell membrane.

#### Cell Internalization

Particles with sizes <10 nm can pass through the cell plasma membrane, called particle internalization (Kumar et al., 2017). Moreover, ZnO-NPs can be transported into the cytoplasm (Manna, 2012). In addition, the interaction between ZnO and bacterial cell membranes can enhance the permeability of cell membranes. Once the ZnO-NPs are internalized by the cells, it will inhibit or cut off the metabolic exchange of substances and energy between bacteria and environment, resulting in the death of bacteria (Zhang et al., 2007).

As a photocatalytic antibacterial agent, many researchers attribute their antibacterial mechanism to the cell damage mediated by ROS generated on the surface of ZnO after photocatalysis. However, some studies are showing that ZnO-NPs also have prominent antibacterial activity under dark conditions (Hirota et al., 2010; Leung et al., 2016). Other factors, including size, surface structure and morphology of ZnO-NPs also bring essential impacts on antibacterial activity (Padmavathy and Vijayaraghavan, 2008; Ansari et al., 2013; Ma et al., 2013; Qi et al., 2013, 2014, 2017) and also the bacteria type (Premanathan et al., 2011). Under different light conditions, the primary antibacterial mechanism of ZnO-NPs is different for different types of bacteria. Therefore, to study the primary antibacterial mechanism of ZnO-NPs under different circumstances and the synergistic effects with other mechanisms is the direction of future research.

## Application Prospects of ZnO-NPs

ZnO-NPs is considered as a relatively safe metal oxide with the inherent ability to induce ROS production and lead to apoptosis, and possesses antibacterial, antifungal, and wound healing activities. We summarize the latest application progress of ZnO-NPs based on antibacterial and antifungal properties, including wound healing, antifungal activity, and prevention of caries.

### Wound Healing

Anti-infection ability and skin regeneration are essential aspects of the wound healing process. The excellent antibacterial property of ZnO-NPs and epithelial stimulation effect of Zn^2+^ have been successfully applied in wound dressings (Lansdown et al., 2007). The traditionally used wound dressings of cellulose, chitosan and alginate cannot be used alone due to their low mechanical properties, which can be improved by addition ZnO-NPs (Alavi and Nokhodchi, 2020). The incorporation of ZnO-NPs into electrospinning collagen/chitosan nanofibers apparently improve antibacterial activity and accelerate wound healing process (Sun et al., 2019). The study of Hu et al. (2018) further suggested that ZnO-NPs showed better biocompatibility than that of silver NPs. These preliminary findings provide new drug candidates for the treatment of increasing antimicrobial resistance and infections.

### Antifungal Ability

In addition to its excellent antibacterial activity, ZnO-NPs also have a significant inhibitory effect on fungus. It has been confirmed that ZnO-NPs can significantly inhibit the growth and reproduction of penicillium and mucor, and the morphologies of ZnO-NPs play critically role in inhibition activity (Zeng et al., 2016). In another study, the decoration of ZnO nanorods on graphene nanoplatelets (ZNGs) can affect the development of the primary virulence factor hyphae of fungus and the formation of biofilms, inducing significant fungal death (Graziella et al., 2018).

### Prevent Caries

Streptococcus mutans is the leading cause of caries. During the killing of Streptococcus mutans, conventional antibiotics and fungicides also harm the healthy flora of the mouth and intestines (Jarvinen et al., 1993). So, agents characterized by a notable antibacterial activity and do not develop resistance are now highly requested. The ZnO-NPs has attracted more and more attentions due to their significant antibacterial properties. It is revealed that ZnO NPs could inhibit biofilm formation of Streptococcus mutans (Eshed et al., 2012). The inhibition activity derived from the relevant mechanical damages exerted by ZNGs and showed a ZnO dose-dependent effect (Elena et al., 2016). Therefore, ZnO-NPs has a promising application as a nano-bactericide for dental pathogens, and maybe a very effective method for controlling the growth of Streptococcus mutans and the development of dental caries.

## Conclusion

Herein, we summarize the main antibacterial mechanisms and application prospects of ZnO-NPs. The excellent biocompatibility, photochemical stability, and other characteristics of ZnO-NPs make it suitable for antibacterial. However, there is still a lack of understanding of its antimicrobial mechanism and toxicity issues. Therefore, researchers should focus on the exactly antibacterial mechanism of ZnO-NPs and adopt more advanced biotechnology to obtain more information on the mechanism. On the other hand, the microstructure of ZnO has a significant influence on its photocatalytic and antibacterial properties. We should develop environmentally friendly synthesis methods and surface modification strategies to improve the antibacterial property of ZnO-NPs. Besides, doping with other metals or non-metallic materials to enhance the selectivity for pathogenic microorganisms and reduce the toxic effect of tissue cells might exert the more extensive biomedical potentials for ZnO-NPs.

## Author Contributions

SJ wrote the manuscript. KL and MC conceived the concept of this review. All authors discussed and commented on the manuscript.

## Conflict of Interest

The authors declare that the research was conducted in the absence of any commercial or financial relationships that could be construed as a potential conflict of interest.
